# Investigating the Knuckleball Effect in Soccer Using a Smart Ball and Training Machine

**DOI:** 10.3390/s22113984

**Published:** 2022-05-24

**Authors:** David Eager, Karlos Ishac, Shilei Zhou, Imam Hossain

**Affiliations:** School of Mechanical and Mechatronic Engineering, Faculty of Engineering and Information Technology, University of Technology Sydney, P.O. Box 123, Broadway, Sydney, NSW 2007, Australia; david.eager@uts.edu.au (D.E.); shilei.zhou@uts.edu.au (S.Z.); mdimam.hossain@uts.edu.au (I.H.)

**Keywords:** knuckleball, smart ball, ball sports, football, soccer, Adidas, sport science, sports dynamics

## Abstract

The term knuckleball in sporting jargon is used to describe a ball that has been launched with minimal spin, resulting in a trajectory that is erratic and unpredictable. This phenomenon was first observed in baseball (where the term originated) and has since been observed in other sports. While knuckleball has long fascinated the scientific community, the bulk of research has primarily focused on knuckleball as it occurs in baseball. Following the changes in the design of the soccer ball after the 2006 World Cup, knuckleball and ball aerodynamics were exploited by soccer players. This research examined the properties of a knuckleball in the sport of soccer. We designed and evaluated a system that could reproduce the knuckleball effect on soccer balls based on previous theories and characteristics outlined in our literature review. Our system is comprised of the Adidas miCoach Smart Ball, a companion smart phone app for data collection, a ball-launching machine with programmable functions, and a video-based tracking system and Tracker motion analysis software. The results from the testing showed that our system was successfully able to produce knuckleball behaviour on the football in a highly consistent manner. This verified the dynamic models of knuckleball that we outline. While a small portion of the data showed some lateral deviations (zig-zag trajectory), this erratic and unpredictable trajectory was much smaller in magnitude when compared to examples seen in professional games. The sensor data from the miCoach app and trajectory data from the Tracker motion analysis software, showed that the knuckleballs were consistently reproduced in-line with theoretical dynamics.

## 1. Introduction

Throughout the history of ball sports, there have been constant developments of new techniques to manipulate the ball trajectory to create an advantage over the opposition. This often involves the application of spin to the ball which alters the trajectory of the ball due to the Magnus effect [1,2,3,4]. In the sport of Association Football (Soccer), the ability to curl the ball from both open-play and dead-ball situations (free kicks) has become a staple of the sport and is especially useful when within scoring range of the goal. If the free kick is taken from a range that has the potential to result in a goal scored directly from the kick, the defending team will often form a wall of players to obstruct a direct path to the goal and make it more difficult for the kicker to score. Given these obstacles, players will often apply different techniques when striking the ball to manipulate the trajectory of the ball in an attempt to beat both the wall and the goalkeeper. Some players may choose to attempt to strike the ball with power, while others will strike the ball with spin to curl the ball over/around the wall (the Magnus effect) [5].

Another challenging technique incorporates minimal spin on the ball and is often used to confuse the opposition due to the erratic and random trajectory of the ball. This technique is known as the knuckleball. Knuckleballs are balls which are launched in such a way that there is minimal spin. While a spinning ball travelling through air creates a pressure differential on one side of the ball which in turn creates a force towards the direction of lower pressure, a ball travelling through air with minimal spin does not create a pressure differential—instead the surface conditions and the slow rotation of the ball generate a turbulence that varies the trajectory of the ball in an unpredictable manner [6]. Although knuckleballs are observed at a specific window of velocities often well below the maximum observed velocity observed in the sport, the erratic and unpredictable trajectory makes it difficult for opponents to judge and defend against, which creates an advantage for those who can master the technique.

The term ‘knuckleball’ and the phenomenon itself was first observed in baseball. While it is not exactly known who first developed the technique, it is often attributed to Edward Cicotte [7] as early as 1908. To achieve this effect, he held the ball between his knuckles, which garnered the nickname ‘Knuckles’ for himself, naming the technique ‘knuckleball’ as a result. Knuckleballs have also been observed in both soccer and volleyball [8] and serve the same purpose in challenging the opponent.

The knuckleball was also popular in the early 1990s and 2000s due to Brazilian football star Juninho Pernambucano, but the technique did not really reach global fame until the 2010s when modern football stars, such as Cristiano Ronaldo and Gareth Bale, also used the knuckleball and brought it to the world stage. The difficulty of performing the shot, and difficulty in stopping it, make it quite a spectacle to behold when performed correctly, with Brazilian people describing it as the ‘Pombo Sem Asa’ or ‘Dove Without Wings’ [9]. While knuckleballs have been observed in open play, they are more commonly seen from free-kick situations. This is due to the time afforded to the kicker in free-kick situations to set themselves up and prepare the correct technique required, as opposed to open-play situations where they are seldom afforded this time.

Normally, knuckleball skills are trained by repeated practice with some instructions acquired from experience. Nowadays, advanced technologies have been widely used to explore the techniques of sports. Videos are widely used to record sports actions which can then be decomposed and analysed step-by-step [10,11]. Other players can grasp and learn the techniques in detail by reviewing the analyzed footage. Accelerometers and inertial measurement units are used to measure the force and movement which help to understand the dynamics of a sports activity and the impacts on humans [12,13,14]. We have also explored embedded-sensor-type systems in the past for robotic arm control [15] and human posture-sensing [16], both of which have sports biomechanics implications and the interface with humans. In the study presented in this paper, the sensors are embedded in the soccer ball and there is no human interaction to minimize human error while observing the knuckleball effect. With the help of modern sensor and measurement technologies, numerous studies have been conducted to investigate the knuckleball effect, including the ball trajectory, speed and spin characteristics, air dynamics and striking skills [17,18,19].

In soccer, the design and construction of the balls are first considered where the presence of seams creates an asymmetry of flow around the ball [20], which varies depending on the orientation of the ball, with many papers examining and validating the effect of seams on ball aerodynamics. Additionally, it has been reported that the knuckling effect has been observed on balls and objects without seams, which suggests that there are additional forces or conditions which influence the phenomenon [8].

To conduct our research, we first needed to develop an understanding of the theoretical models of different soccer ball trajectories, especially the knuckleball effect. A phase diagram has been proposed in [21] to categorize soccer trajectories into parabolas, straight lines, zigzag and spiral trajectories. It illustrates that ball trajectories are affected by the ratio of drag force and gravity, and the ratio of ball spin and speed. Knuckleballs have the characteristics of low spin and high speed. In [22], two cameras were used simultaneously to record the knuckleball trajectory which comprised three-dimensional coordinates of the ball positions. Significant fluctuation was found around the top of the ball trajectory. The air drag coefficient of a soccer ball was investigated in [23] considering two cases: dropping the ball from a high building without any initial spin and speed, and a goalie punt to generate projectile motion. The results indicated that outdoor wind and ball asymmetry had a considerable impact on the ball trajectory; thus, it was recommended that the experiment should be conducted indoors, using high-surface-quality balls.

Another important aspect of knuckleballs is the striking of the ball and the techniques and resultant dynamics associated with this [24,25,26]. As demonstrated in [27], to achieve the knuckleball, synchronized pelvis roll and yaw rotation is essential. It was also found that pelvis roll and yaw rotational angles during a knuckleball kick were smaller than these during a normal instep kick. The motions of the lower limbs and the pelvis were further researched in [28] which concluded that the straight foot trajectory in the striking phase was important to obtain the knuckleball effect. Comparing the lower limb joint torque among different soccer kicking motions, it was found that the adduction torque of the hip joint decreased for curve kicks, while it increased for knuckle kicks [29]. The striking points of three typical soccer kicks were researched in [30] with the results showing that the striking point of a straight kick was located at the center area of the instep, the striking point of a curve kick was located at the low area near the inside of the foot, and the striking point of a knuckle kick was located near the foot joint.

Contact time is an important factor in ball dynamics and is determined by the location and geometry of the foot strike (which part of the foot is used and the shape and flexion of the foot) and the characteristics of the ball, such as the material, surface roughness and pressure (e.g., inflation pressure and over-pressure). Ball characteristics are governed by regulations set by the governing body, FIFA, which outlines the minimum requirements and acceptable tolerances for the ball [31].

Research by [32] found a weak negative relationship between contact time and ball velocity. Curled shots, or shots with high levels of spin, were found to have longer contact times as kickers attempted to maximise contact time with the ball to impart spin to it. Conversely, straight shots were found to have much shorter contact times as the kicker sought to minimise contact time to minimise any spin on the ball [9]. Contact time for knuckleball shots would be most similar to straight shots as the kicker tries to hit the ball with minimal spin but different speed. It has been reported that some kickers will position the ball such that they will strike the bladder valve to minimise contact time. This area of the ball is relatively rigid in comparison to the rest of the ball due to the valve itself, and, as such, this face of the ball would deform to a lesser degree than other faces of the ball, thus minimising contact time.

Available research has shown that successful knuckleball shots require very precise striking of the ball and movements of the hips and ankle. To minimise the spin placed on the ball, practitioners aim to strike the ball through the centre of mass. It was found that the angle of strike for a knuckleball was 3.3 ± 0.9°, which was significantly smaller than for straight or curled shots (19.3 ± 2.4°, 32.6 ± 3.9°, respectively) [33]. Additionally, it was determined that the characteristics of the strike for knuckling shots were more similar to curled shots than straight shots with the ankle posture and shape being flexed in similar ways at the point of impact. An analysis of the maximal torque exerted by the knee extension and hip rotation found that, while all shots had similar peak values for knee extension, the peak values for hip rotation for knuckling shots were the highest out of all the shots, (67.2 ± 6.0 Nm against 39.1 ± 3.4 Nm and 53.1 ± 5.8 Nm for straight and curled shots, respectively). This is indicative of a tendency to strike the ball with the heel pushed out towards the inside of the foot when striking a knuckling shot [25].

From this, we can see the technical requirements of the shot executed by a player which contributes to the rarity and exclusivity of the knuckleball shot in soccer. In theory, hitting the ball along the centre of mass would ensure that the ball has no spin. However, in practice, this is not the case due to the small margin of error for the strike zone. Additionally, in an in-game situation, a human wall is erected to prevent a direct shot on goal. This means that players are required to hit the ball slightly under the centre of mass to get the ball up and over the wall. The small angle of strike leaves little room for error, with errant strikes applying some amount of spin to the ball, potentially resulting in the ball going over the bar and missing by a large margin.

This paper aims to present a comprehensive exploration of the knuckleball effect using both theoretical and experimental methods. First, the knuckleball characteristics and the main influencing factors that contribute to the knuckleball effect are explained in detail. Then, the experimental set up is described with a ball training machine and the Adidas miCoach Smart Ball. By adjusting the ball training machine actuators with different striking positions and power, knuckleballs were successfully realized in this research. In addition to use of the ball training machine, which provided ball-strike information, this research also adopted high-speed cameras to record the ball trajectory. A zig-zag trajectory was clearly observed for knuckleballs, while a smooth trajectory was observed for non-knuckleballs. Based on the fact that a knuckleball normally has low spin, investigation of the speed and spin information provided by the Adidas miCoach Smart Ball also enabled understanding of the characteristics of knuckleballs. With the help of the ball training machine, Adidas miCoach Smart Ball and cameras, the knuckleball was analysed in depth, benefiting understanding of the origin and characteristics of, and factors influencing, knuckleball.

## 2. Methods

### 2.1. Design Requirements

When considering the development of our system in relation to our research question, we outlined several design requirements that we followed strictly throughout the research project. The design requirements were as follows:

- Understanding of theoretical models of the knuckleball effect;
- Consistent method of launching the soccer ball;
- Programmable method of launching the soccer ball;
- Capability to track the trajectory of the ball;
- Capability to accurately track the kinematics of the ball; and
- Accurate data collection methods by minimizing human input.

**Figure 1:**
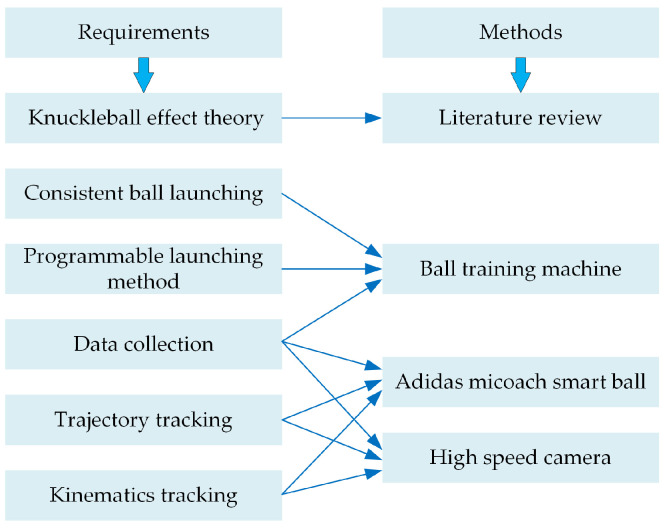
Research requirements and methods.

Based on these design requirements, we firstly acquired a comprehensive understanding of the knuckleball effect through an extensive literature review and analysis of existing theoretical models. We then designed the physical system based on reducing human error and prioritizing accuracy and consistency. For this, we opted for a computer-based system which consisted of a ball training machine that had programmable functions and the Adidas miCoach Smart Ball which had internal sensors that could produce kinematic data of the ball. We also supplemented the smart-ball data with video-based data collection of the ball’s trajectory through implementation of a multi-camera system on the field. Figure 1 is a diagram that summarises the requirements and the corresponding methods used in this research.

### 2.2. System Design

The system presented in our research consisted of the Adidas miCoach Smart Ball and companion smartphone app, Ball-Training Machine (BTM) for launching the ball, a dual-camera setup and Tracker software for visualizing the ball trajectory.

**Figure 2:**
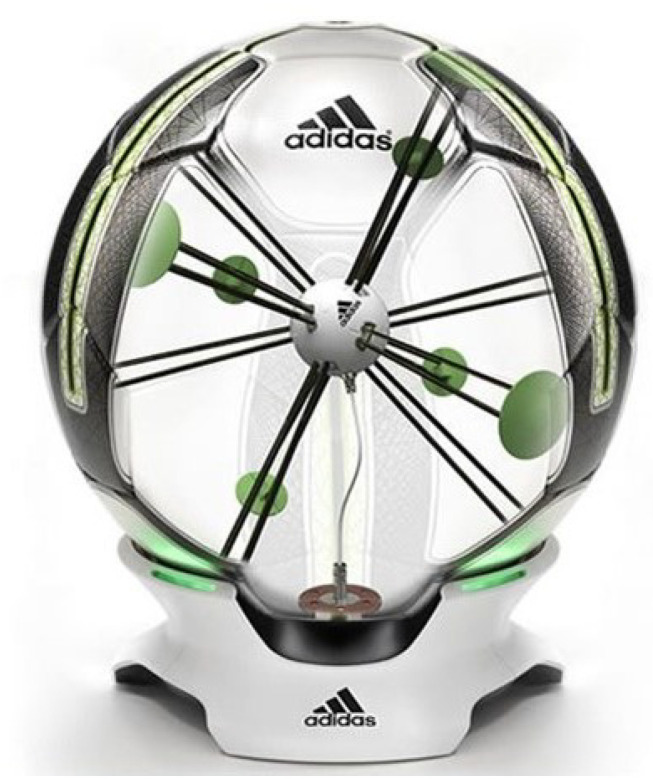
Adidas MiCoach Smart Ball [34].

The Adidas miCoach Smart Ball [34] is depicted in Figure 2 and is a training tool developed by Adidas for training kicking from dead-ball situations, such as free kicks and penalties. Along with the companion application for mobile devices, the ball provides the users with data on power, spin, strike point and trajectory which can be used to train players by providing them with feedback. Based on this feedback, players can adjust their technique accordingly.

The ball is constructed to meet Fédération Internationale de Football Association (FIFA) Regulations for balls in terms of weight and size and features a thermally bonded conventional thirty-two-panel design. Internally, the traditional ball bladder has been modified to house a six-axis accelerometer sensor package. The sensor package is suspended centrally within the bladder by twelve sets of kevlar loops. The sensor system is shown in Figure 2.

Once the ball has been struck by the user, the information received from the sensor is processed and output to a connected Bluetooth compatible smartphone device. It is assumed that the added sensor package and suspension system will have no impact on ball performance in terms of movement and feel and that the ball should behave the same way any other ball would under the same circumstances. The Smart Ball is used to provide additional data from testing that would usually be difficult to measure specifically, such as the spin and strike location. This data, in conjunction with the visual data obtained from the high-speed cameras, was analysed to provide an understanding of the knuckleball trajectory.

**Figure 3:**
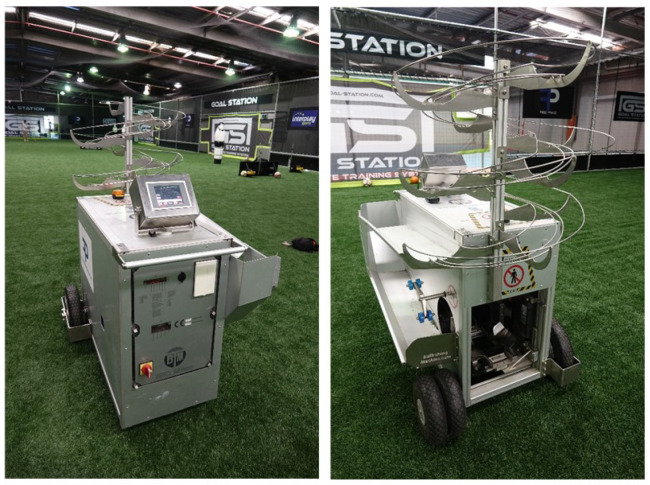
Ball Training Machine (BTM) by Ball Training Machine APS.

Due to the difficult nature of performing a knuckleball, let alone performing them consistently enough to record data, it was deemed necessary to use a programmable machine that would be able to produce the effect reliably. As a result, we approached First Phase Football based in Seven Hills, New South Wales, Australia. First Phase Football allowed us to use their launcher called the Ball Training Machine which is manufactured by Ball Training Machine APS, a company based in Denmark. The BTM is depicted in Figure 3. The launcher was both designed and manufactured in Denmark. With the machine being made to order, this was one of the few in the world and the only known one in Australia.

**Figure 4:**
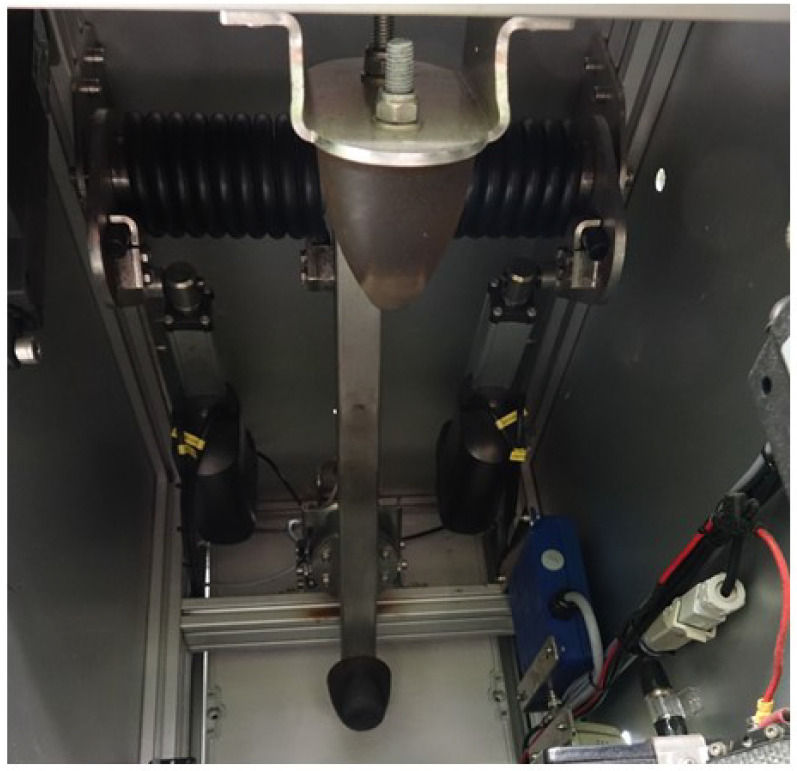
Ball Training Machine kicking mechanism.

**Figure 5:**
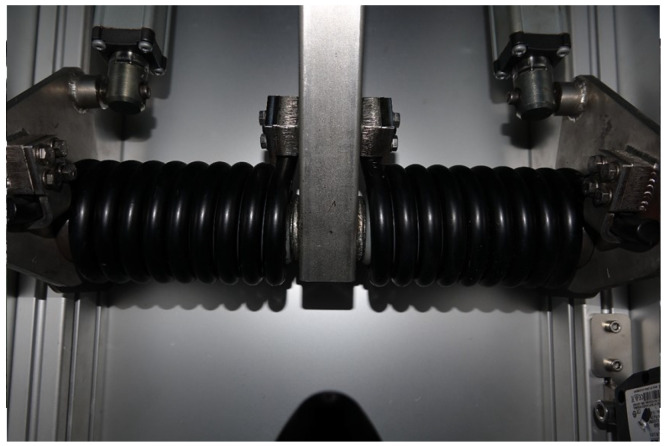
Ball Training Machine—spring mechanism.

The design of the BTM was aimed at simulating striking the ball with the foot, as opposed to launchers which utilise rollers to launch the ball. Figure 4 shows the striking mechanism/arm and the attachment used to strike the ball. Figure 5 illustrates the design principle of the striking mechanism. The force of the arm is varied via actuators attached to a spring and can be varied depending on what is input into the system.

**Figure 6:**
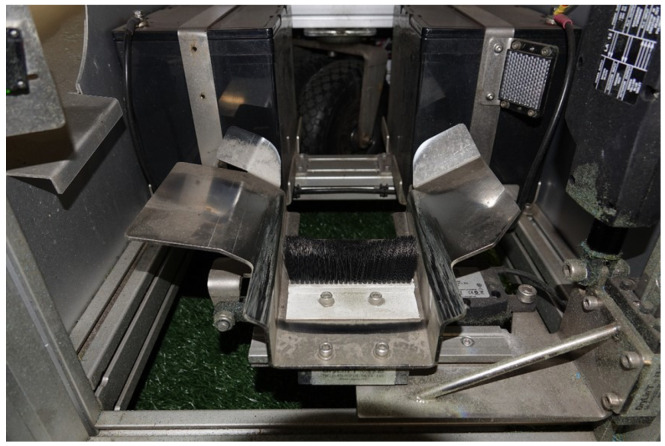
Ball Training Machine—loading tray for balls.

**Figure 7:**
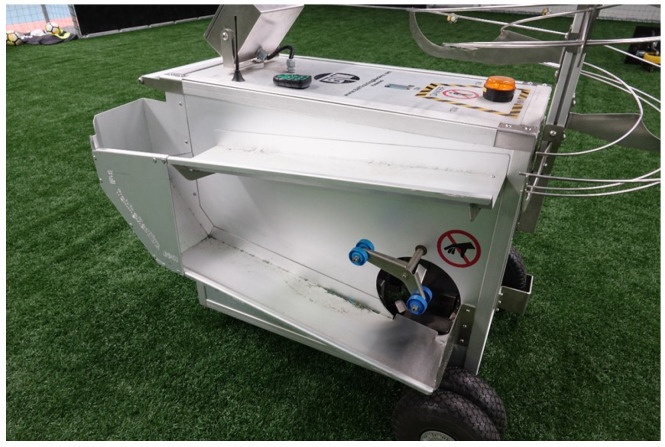
Ball Training Machine—ball-feeding mechanism.

Figure 6 shows the tray in which the ball sits before it is kicked by the launcher. The tray sits on rails mounted to linear actuators to allow for the lateral placement of the ball to be varied which is what allows the launcher to generate spinning shots. The BTM also features a rail and feeding mechanism which can store up to 10 balls for continuous operation, minimising the need to manually reload the ball after every usage. This was not utilised for this experiment as the Smart Ball was used for all shots for consistency and manually reloaded for each shot. Figure 7 shows the rail and feeding mechanism of the BTM.

**Figure 8:**
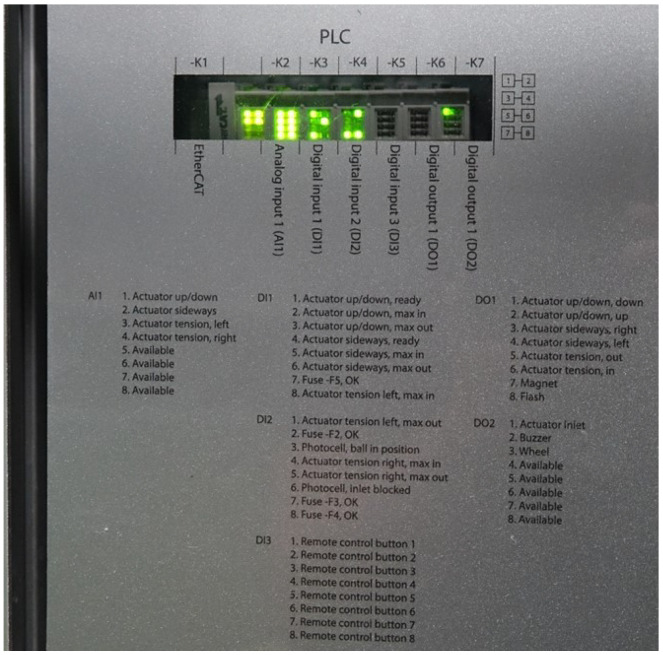
Ball Training Machine—programmable logic controller functions.

The BTM utilises a programmable logic controller (PLC) system to control various actuators and sensors to produce the required kicked based on user input. Figure 8 shows the back panel of the BTM. This panel has a window to the PLC housing allowing viewing of the board, as well as a list of the inputs and outputs of the board, and the function of each of them.

**Figure 9:**
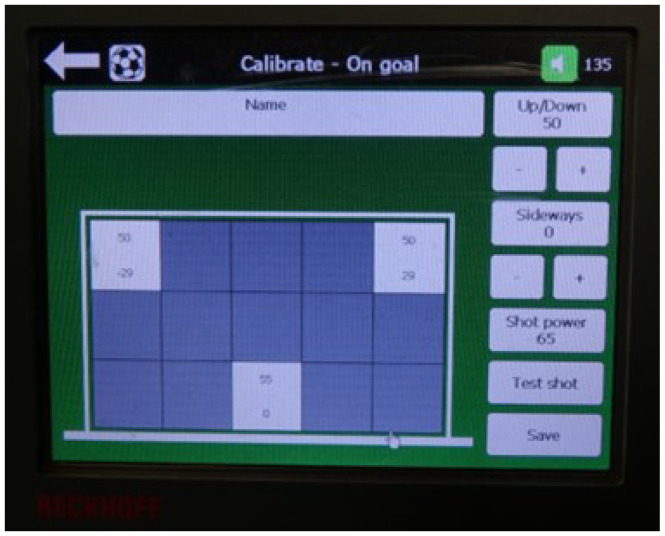
Ball Training Machine—On Screen Menu.

The user input is performed via touchscreen display as shown in Figure 9. The system inputs are on a scale of 0–100. Users can choose to input specific values and settings or utilise the various programmed combinations developed by Ball Training Machine APS. The inputs and their function are as follows:

- Up/down—height of the ball where 0 is a ground shot/pass and 100 is the maximum vertical shot/pass;
- Sideways—spin, where to the right is a positive input value and to the left is a negative input value; and
- Shot Power—power of the shot (speed).

The values of these inputs are used to vary the position of the appropriate actuators to vary the strike location of the striking mechanism to achieve the desired kick.

The BTM can perform a variety of shots, ranging from curled and straight shots to various shots on a goal, with a high degree of accuracy. Users can set the BTM to produce a variety of shots or passes, both with and without spin, to simulate various aspects of the game.

Spinning shots are generated by changing the position of the ball tray using the linear actuator. This allows the arm to strike the ball off-centre and impart spin to it. For straight shots, the tray remains in its original position and the arm simply hits the ball. The magnitude of spin and the speed of the shot are determined by the power and strike location of the arm which is set by the user using the input interface.

## 3. Results

### 3.1. Experimental Setup

**Figure 10:**
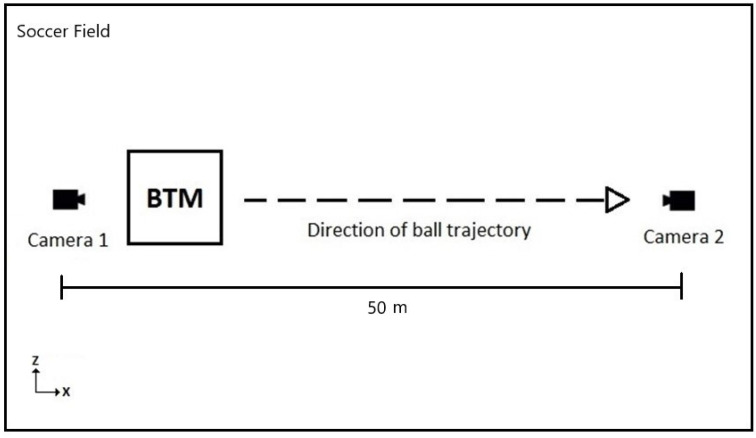
Experimental setup for kick trajectory analysis.

We conducted our experiments at the First Phase Football arena located in Seven Hills, NSW, Australia. The venue offers training facilities for soccer athletes of all levels to develop their skills. The facility features a 1000 m${}^{2}$ training space turfed with synthetic grass and eight full-sized goals designed to replicate various scenarios on a football pitch. For analysis, we used the Adidas miCoach Smart Ball for sensor data that was transmitted to the partnered smartphone app, a two camera setup for visualizing the ball’s trajectory, and a ball training machine for programming the launch settings. The experimental setup can be seen in Figure 10.

To observe the full trajectory of the ball, the BTM was set up to shoot across the testing area, as opposed to shooting towards a goal. The distance between the cameras was approximately 50 m. The motion of the ball was recorded using two high-speed Sony RX10 III cameras. The footage was captured at a rate of 500 fps and a resolution of 1920 × 1080 pixels (×10 slow motion). Cameras were placed both behind and in front of the launcher, as shown in Figure 10, to observe the lateral deviations of the ball. As there was no external trigger available, each camera had to be manually triggered to record the data. We used Tracker video motion analysis and modelling software to visualize the trajectory of the ball in the videos. Tracker [35] is an open-source video analysis and modelling tool built on the Open-Source Physics (OSP) Java framework. Its main features include object tracking with position, velocity and acceleration, centre of mass tracking and vector analysis.

While spinning and straight shots could easily be generated, or were already pre-programmed into the system, the settings had to be adjusted and calibrated to achieve the required knuckling effect. It was found that the BTM settings which yielded a knuckleball trajectory were a vertical of 50 and a power of 55 which equated to an average speed of approximately 94 km/h and 65 rpm as measured by the Adidas miCoach Smart Ball.

**Figure 11:**
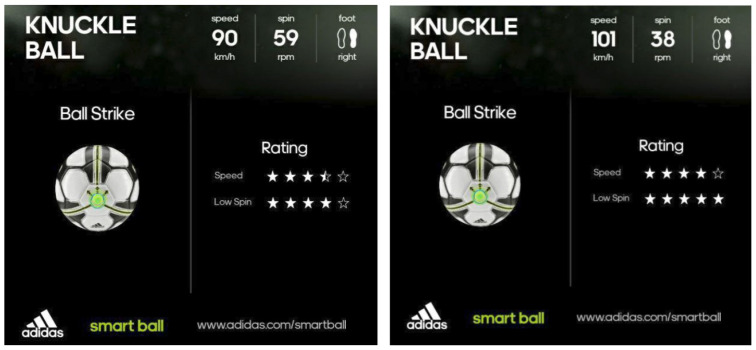
Example display from miCoach Smart Ball app.

The Adidas Smart Ball was used for all the tests conducted and was manually retrieved and placed into the ball tray. The Smart Ball has a circumference of 690 mm, diameter of 220 mm, mass of 442 g and density of 79.46 kg/m${}^{3}$. The ball was paired with an Android smartphone and the companion application was used to retrieve data on ball spin and speed. An example of the visual display of data from the companion app is depicted in Figure 11.

### 3.2. Experiment Outcomes

The aim of our experiment was to analyze the dynamics involved in producing a knuckleball trajectory on a standard soccer ball. We conducted testing over numerous sessions and recorded 60 samples in total, which equated to 60 ball launches from the BTM. From the 60 samples recorded, 42 of the shots could be used. This was due to errors from mistiming the triggers of the cameras where the trigger was depressed too early or late, resulting in footage which did not capture the full flight of the ball.

To analyse the data the footage was input into Tracker motion analysis software [35]. Due to the positioning of our cameras and the nature of the footage, with the subject moving away/towards the camera, the software was unable to be used to measure distances within the footage, and, instead, the software was used for trajectory visualisation purposes. Additionally, Adobe Premiere was used to process the footage by modifying the playback speed for comparative purposes.

**Figure 12:**
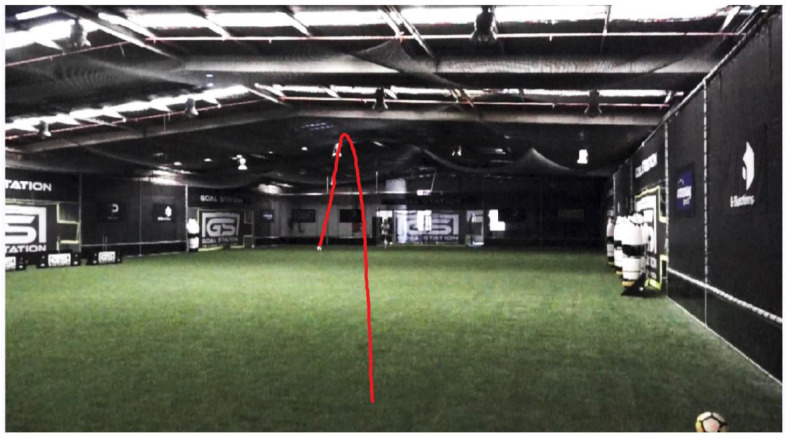
Example of typical trajectory of soccer ball.

While the conditions for all shots were kept identical, no two samples collected had the exact same trajectory. Figure 12 shows an example of the common trajectory recorded (marked by the red trail). In these cases, the ball travelled in an initial pseudo-straight trajectory before veering to the right. This is considered the typical trajectory.

**Figure 13:**
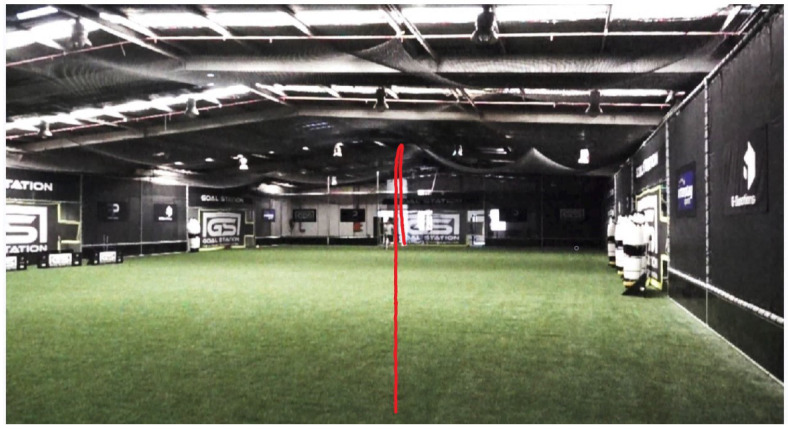
Example of knuckle ball trajectory of soccer ball.

Several data samples collected exhibited lateral deviations (zig-zag trajectory). Figure 13 shows one example of the zig-zag trajectories achieved by the BTM (marked by the red trail). In instances where a zig-zag trajectory was observed, the lateral deviations of the ball were between 0.5 to 0.7 of the ball’s diameter (110 to 154 mm) with one to two changes in direction observed. Feedback from the Smart Ball showed that the speed of these shots ranged from 79 to 109 km/h and averaged 94 km/h. The ball spin (measured in rpm) ranged from 38 to 114 rpm and averaged 65 rpm.

From the 42 samples, we considered five random samples for the typical and knuckleball trajectories. The results for typical trajectory samples are tabulated in Table 1. The results for the knuckleball trajectory samples are tabulated in Table 2. The tables include ball speed and ball spin as measured by the app, as well as the speed and low-spin rating (rated out of five) which was determined by the miCoach app. The means of all the samples were also calculated and are presented in their respective tables.

## 4. Discussion

### 4.1. Data Analysis and Evaluation

Based on the results observed across all samples, the data was congruent with the theory found in the literature review stage of the research. Testing appeared to confirm that the thirty-two-panel design of the miCoach ball suffered minimal variances in trajectory given varying orientations of the ball. While the numerical value of the forces on the ball could not be measured, a visual inspection of the collected data was able to confirm this. As noted in the results section, while no two trajectories were the same, the majority of them followed a similar path.

The speed values of the data collected ranged from 79 to 109 km/h and averaged 94 km/h. A level of the knuckling effect was found for these shots (a minimum of one lateral direction change), with some shots exhibiting more than one directional change. This was congruent with research which found that, depending on the soccer ball used (from 2006 onwards), knuckling would begin to occur at speeds of approximately 80 km/h and higher.

At full speed, the total time for one of the recorded kicks was only 1.46 s over an approximately 50 m distance. This was far greater than the average shooting distance of the sport. In an in-game scenario, this would be the equivalent of a shot from half-way of the soccer field. Shots can range from anywhere between 0 to 35 m from the goal with typical long-range shots ranging from 15 to 30 m. While knuckleballs have comparably smaller lateral deviations than spinning kicks, the erratic nature of the kick can be enough to confuse a goalkeeper, especially given the short amount of time they may have to react.

The companion application of the Smart Ball rates each kick on a scale of five, stars as shown in Table 1 and Table 2. It defines an ideal knuckleball as one that experiences minimal spin (40 rpm or lower) and maximum velocity (113 km/h or higher). Throughout testing, we attempted to achieve a five-star rating for both speed and spin; however, in practice, it proved to be extremely difficult to achieve this, given the need to accurately strike the ball with sufficient power while also minimising spin. The best result was one that achieved a speed of 101 km/h and and a spin of 38 rpm (five and four stars, respectively).

The knuckleball shot was made famous by Cristiano Ronaldo, one of the most skilled soccer players in the history of the sport. Executing the skill has proven to require advanced technique, and, even with controlled methods, it is not a simple task. For beginners, it would be easier to focus on striking the ball properly and minimising the spin rather than striking for power.

Figure 12 and Figure 13 show the typical trajectory data collected by the Tracker motion software. From Figure 12, we can observe a typical trajectory for a ball (no knuckle effect). The figure shows that the typical shot causes the ball to veer to one side. This could be attributed to the higher spin experienced on the ball as well as the striking location of the ball. This is a much more common technique in football matches. Figure 13 shows that the knuckleball shot exhibited a more linear path in comparison. Although there were lateral deviations observed, the ball tended to stick to a more direct trajectory. The straighter pathway can be attributed to the low spin produced by the knuckleball shot. These observations from the Tracker software verify the functions of the ball training machine and results observed from the miCoach app.

### 4.2. Comparing Kick Types

When comparing different techniques of kicks, there are numerous advantages and disadvantages for each style. Based on numerical calculations and physical data collection, it was found that a knuckleball performed in soccer can exhibit between one and two lateral changes in direction over a range of 30 m, which can easily leave the goalkeeper wrong-footed and unable to make a save. However, this unpredictability is also a disadvantage, as the trajectory cannot be fully controlled by the kicker and the ball may simply fly out with minimal pressure on the goalkeeper. Additionally, during testing there were attempts to produce two identical shots, but no two knuckleballs produced were the same, further exemplifying the randomness of knuckleball trajectories. Another factor is the difficulty in performing a knuckleball shot. While technological innovations have made it easier to perform knuckleballs, the technique has only been mastered by a few professional footballers.

Alternatively, players may elect to utilise a curled or driven (straight) kick. These types of kicks are more controlled in comparison, but again have their own advantages and disadvantages. Comparatively, these kicks are much easier to perform, and are staples of the game that even amateur athletes are expected to have grasped. The trajectory can be controlled depending on the amount of spin placed on the ball and the speed of the kick, which can be adjusted depending on the range of the kick. Additionally, curled and driven kicks have been observed for a range of speeds relatively higher than the range of speeds typical of a knuckleball. While these kicks travel relatively faster than knuckleballs, their trajectories are easy to understand and judge and defending them depends on judging the direction of the trajectory and reacting quickly enough.

It cannot be definitively said which technique is better than the other as each has its advantages and disadvantages. While the knuckleball can potentially be devastating, it is essentially a gamble on whether or not the shot will have the intended result. On the other hand, curled and driven shots can be considered to be more accurate as the player can aim the shot and try to place the ball away from the keeper, though the relatively straightforward trajectory means that they can be stopped more easily, subject to keeper judgement and reflexes. Ultimately, the shot selection comes down to player preference and skill, as well as the circumstances of the free kick, with the best free-kick takers able to adapt and select the right shot for the situation.

### 4.3. Limitations and Future Improvements

While some knuckling was achieved, the magnitude was much smaller than has previously been observed in both professional and amateur environments. These results can be attributable to numerous factors which limited the ability to produce an exaggerated knuckling effect.

The choice to use the Adidas miCoach Smart Ball for testing limited the ability to achieve an exaggerated knuckling trajectory. This was due to the technology used to construct the ball. As explored earlier, the design of the panels on a soccer ball has an impact on the trajectory due to the aerodynamic variances on the faces of the ball. A ball using the traditional thirty-two panel design minimises these variances due to the relatively symmetrical faces of the ball due to the consistent design.

Furthermore, the decision to conduct testing indoors also limited the ability to achieve an exaggerated knuckling effect. While an indoor facility created a controlled environment and eliminated various weather factors, this may have also hindered the ability to achieve the desired knuckling. The addition of wind would add a factor of randomness to the trajectory with the variances in trajectory depending on the weather conditions. It is believed that the magnitude of knuckling will vary should testing be moved to an outside space with various weather conditions.

Additionally, the design of the launcher may have affected the quality of the knuckling seen. The feeding mechanism of the launcher where the ball rolls from the rail into the tray can result in the ball not sitting in the tray uniformly each time. While most of the results collected had a similar non-identical trajectory, there were some outliers which could possibly be explained by the variances in the final position of the ball prior to being launched from the launcher. However, due to the design of the launcher, this cannot be confirmed. The use of a launcher with a different design could possibly produce different results depending on the mechanics and design, such as a design using rollers to launch the ball.

## 5. Conclusions

Our results demonstrated that we were successfully able to reproduce and analyze the characteristics of the knuckleball effect in soccer. The data evaluated from the miCoach Smart Ball showed that knuckleball shots maintained a low spin (rpm) with high speeds over short distances. The motion data observed through the Tracker software further confirmed the randomness of the knuckleball trajectory in comparison to a typical trajectory kick.

The growing prevalence of knuckleballs in soccer can be explained as a culmination of numerous factors. While the technique has become more popular in recent years, due to high profile players, such as Cristiano Ronaldo and Gareth Bale, adopting it, it remains a rare sight due to the difficulty in performing the shot and the benefits of other, much simpler, types of shots (e.g., curled and straight/driven shots). Obtaining an exaggerated knuckling effect requires numerous conditions to be satisfied. In addition to the absence of spin, the ball must be launched/struck at a speed which coincides with the drag crisis of the ball (the critical speed). Recent changes in the materials, design and construction of the ball has made the ball lighter and more spherical, resulting in the critical speed coinciding with the typical speeds of shots and long-range passes. However, the precise angle of strike, and the technique required, means that the knuckleball remains a rare sight in soccer.

While experimental testing was able to produce satisfactory results and to support most of the theoretical data collected, it is believed with further testing that a highly exaggerated knuckling effect can be achieved given some modifications to the testing procedure.

## Figures and Tables

**Table 1:** Dynamics of typical trajectory of soccer ball trial.

Trial	Ball Speed (km/h)	Ball Spin (rpm)	Speed Rating (/5)	Low Spin Rating (/5)
1	99	64	4.0	4
2	90	62	3.5	4
3	90	59	3.5	4
4	90	61	3.5	4
5	89	63	3.0	4
Mean	91.6	61.8	3.5	4
SD	4.16	1.92	0.35	0

**Table 2:** Dynamics of knuckle ball trajectory of soccer ball trial.

Trial	Ball Speed (km/h)	Ball Spin (rpm)	Speed Rating (/5)	Low Spin Rating (/5)
1	95	47	3.5	4.5
2	101	38	4.0	5.0
3	99	47	4.0	4.5
4	79	53	2.5	4.5
5	98	58	4.0	4.0
Mean	94.4	48.6	3.6	4.5
SD	8.88	7.50	0.65	0.35

## Data Availability

The data presented in this study are available on request from the corresponding author.

## Abbreviations

The following abbreviations are used in this manuscript:

BTM	ball training machine
FIFA	Fédération Internationale de Football Association
FPS	frames per second
PLC	programmable logic controller
